# Supplementary material for: Forecasting extreme stratospheric polar vortex events
Source: Nat Commun. 2020 Sep 15;11:4630. doi: 10.1038/s41467-020-18299-7 (PMC7492229; doi:10.1038/s41467-020-18299-7)
Supplement: Supplementary file 1 — Supplementary Information [file 41467_2020_18299_MOESM1_ESM.pdf]

Supplementary Information:

Forecasting Extreme Stratospheric Polar Vortex Events

Gray et al.

Supplementary Note 1.

Dynamics of Sudden Stratospheric Warmings

Observations of the winter stratosphere have improved substantially since the late 1970s with the advent of satellite observing platforms, along with a growing understanding of stratospheric dynamics[1]. The stratospheric polar vortex develops in response to seasonal changes of equator-to-pole temperature gradients in the stratosphere but is intermittently disturbed by upward propagating planetary-scale (Rossby) waves from below, generated by land-sea heating contrasts and the disruption of tropospheric mid-latitude jets by major mountain ranges. These large-scale waves can be refracted, reflected or absorbed depending on the background flow through which they propagate. Decreasing air density with height means that the wave amplitudes grow exponentially as they travel upward, so they can also break like ocean waves on the shore and in doing so transfer easterly momentum to the background flow thus slowing down the ambient westerly vortex flow.

Sudden warmings occur as the result of a particularly large amplitude Rossby wave event, sometimes involving resonant behaviour[2] or the gradual attrition of wave-breaking events over an extended period[3,4]. The vortex moves away from the polar regions during a displaced (wave-1) warming and is split into two separate vortices in a split (wave-2) warming[5-7]. Provided there is sufficient time before the end of winter the vortex westerlies are able to re-generate, but the anomalously weak winds in the lower stratosphere can linger for up to 60 days, with associated impacts at the surface.

The 2008/9 Stratospheric Sudden Warming

2008/9 provides a classic example of a mid-winter split vortex warming[8-10]. The polar vortex does not strengthen smoothly throughout the winter (Figure 1a); it is intermittently disturbed by upward propagating wave packets from the troposphere that serve to weaken the flow, for example in early November and again in early December, until finally a major warming is established towards the end of January. Easterly winds are initiated in the upper stratosphere and then extend deep into the lower stratosphere, and the latter remains disturbed for several months. Notice that the wave burst in early December (Figure 1a) is almost strong enough to reverse the winds at the upper levels, as indicated by the presence of a zero-wind contour at ~0.5 hPa in early December, but this upper level wind reversal does not extend into the lower stratosphere to achieve a full (major) warming.

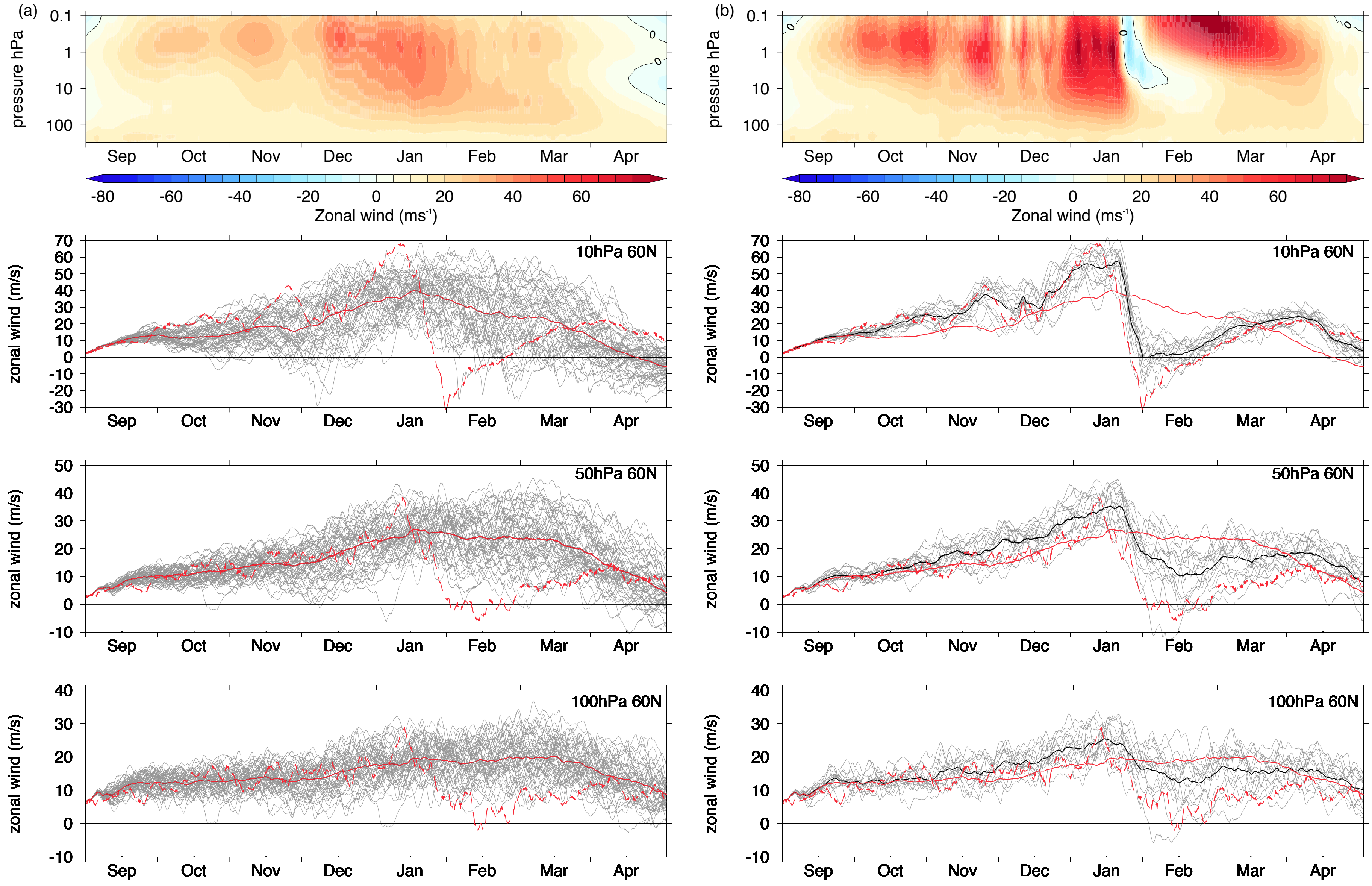

Supplementary Figure 1. 2008/9 Control Run and UpStrat Experiment . Zonally averaged zonal winds (ms^{-1}) at 60°N. **(a)** 2008/9 Control Run in which no relaxation was imposed. **(b)** 2008/9 UpStrat Experiment, in which **u,v** and T were relaxed towards ERA-Interim data above the 5 hPa level at all latitudes. Top row shows the ensemble-mean evolution while the lower panels show the evolution of individual ensemble members (grey lines) at selected pressure levels. Dashed red lines show the ERA-Interim data. Red solid line shows the Control Run ensemble-mean. Solid black line in (b) shows the UpStrat ensemble average.

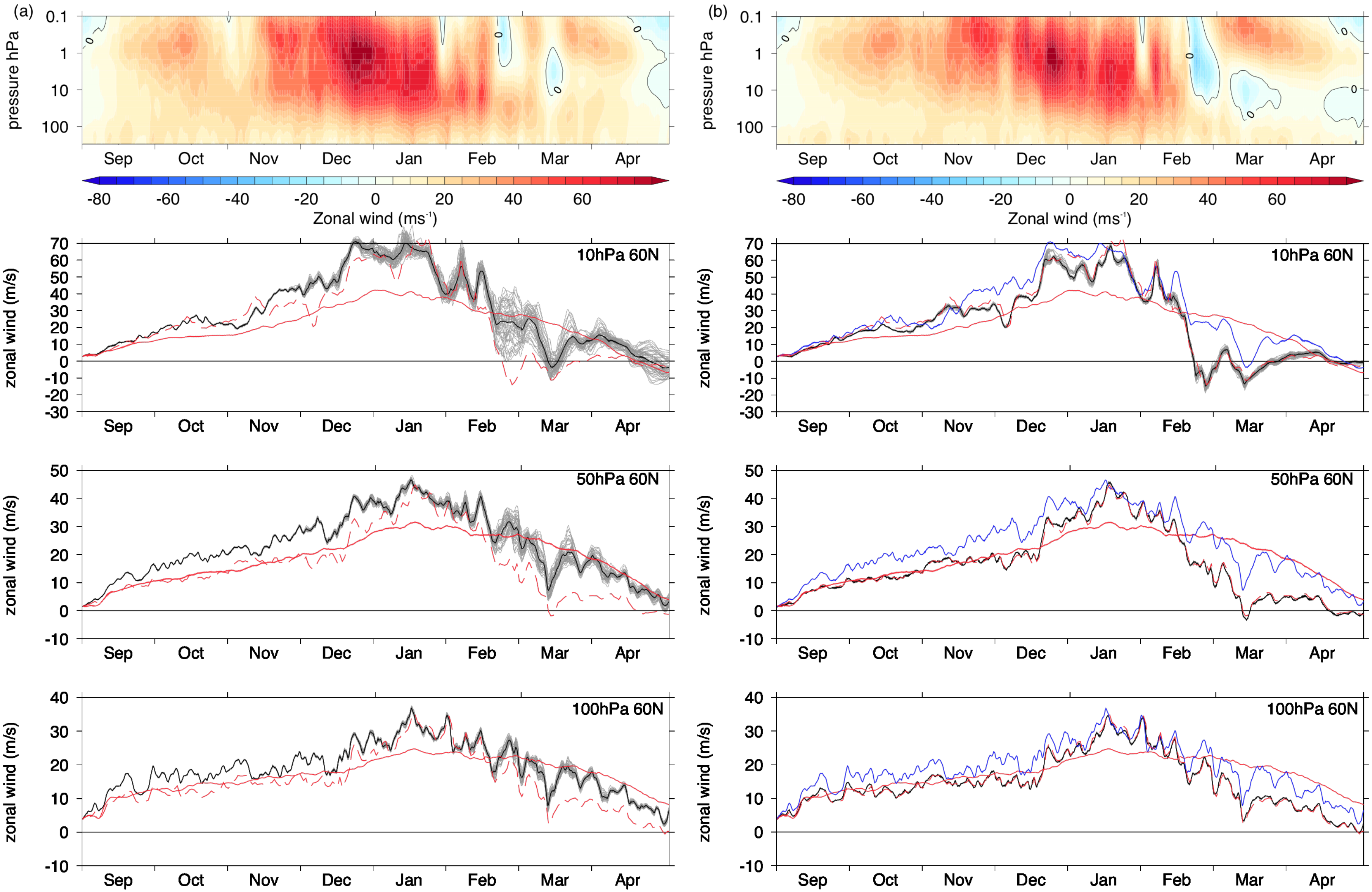

Supplementary Figure 2. **The 1988/9 AllTrop and AllTrop-UpStrat-Eq Experiments.** Zonally averaged zonal winds (ms^{-1}) at 60°N. **(a)** 1988/9 AllTrop experiment in which **u,v** and T were relaxed towards ERA-Interim data throughout the troposphere. **(b)** 1988/9 AllTrop-UpStrat-Eq, identical to All-Trop except for additional relaxation of u to ERA-Interim data above 5 hPa and between 0-10°N. Top row shows the ensemble-mean evolution while the lower panels show the evolution of individual ensemble members (grey lines) at selected pressure levels. Thick black line shows the ensemble average. Dashed red lines show the ERA-Interim data. Red solid line shows the Control Run ensemble-mean for comparison. Blue line in (b) shows the AllTrop ensemble-mean for comparison.

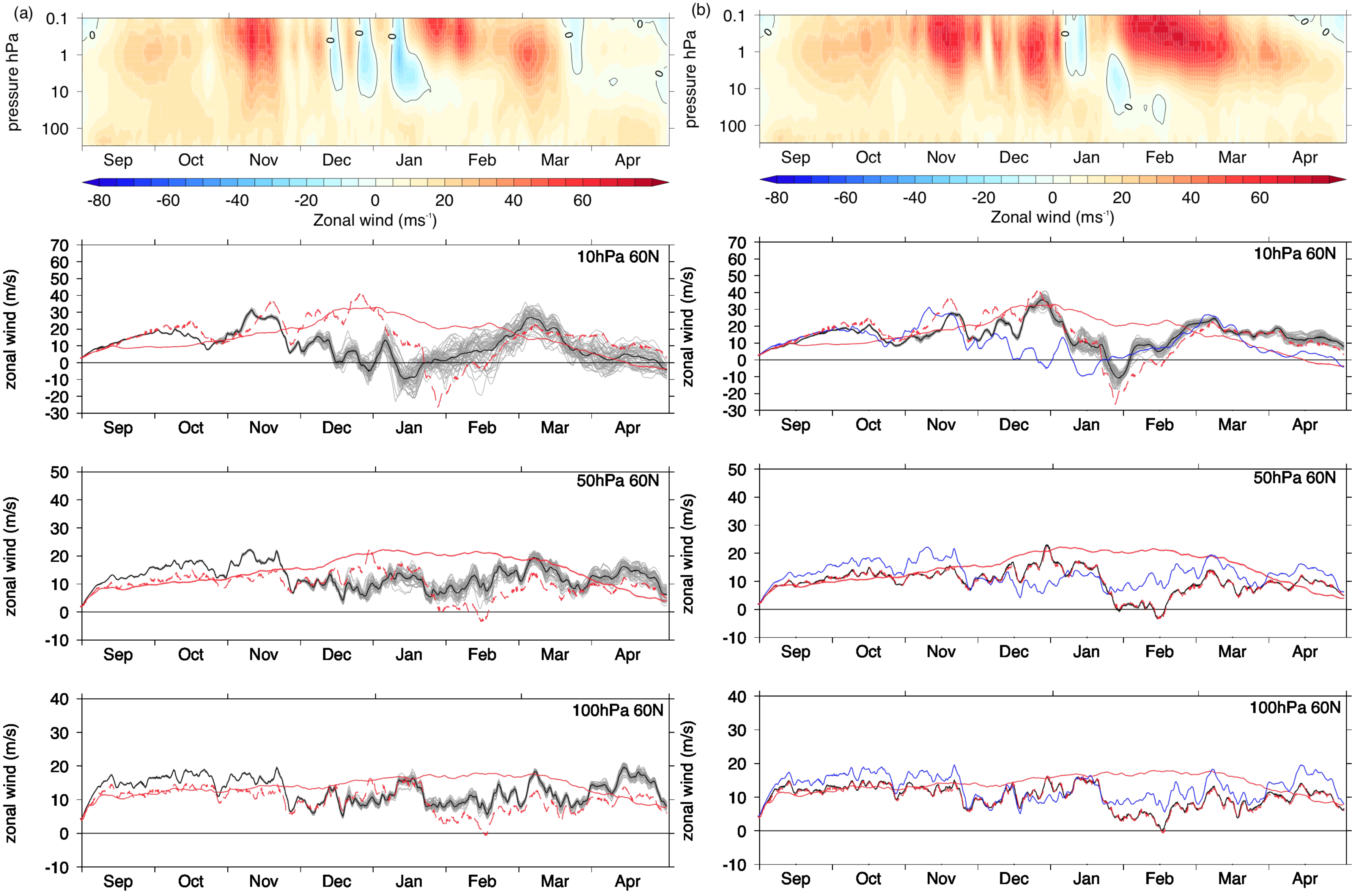

Supplementary Figure 3. The 2005/6 AllTrop and AllTrop-UpStrat-Eq Experiments. Evolution of zonally averaged zonal winds (ms^{-1}) at 60°N. **(a) 2005/6** AllTrop experiment in which **u,v** and T were relaxed towards ERA-Interim data throughout the troposphere. **(b) 2005/6** AllTrop-UpStrat-Eq, identical to All-Trop except for additional relaxation of **u** towards ERA-Interim data above 5 hPa and between 0-10°N. Top row shows the ensemble-mean evolution while the lower panels show the evolution of individual ensemble members (grey lines) at selected pressure levels. Thick black line shows the ensemble average. Dashed red lines show the ERA-Interim data. Red solid line shows the Control Run ensemble-mean for comparison. Blue line in (b) shows the AllTrop ensemble-mean for comparison.

Supplementary Figure 4. 2008/9 E-P Flux Divergences. Pressure (hPa) versus latitude plots (30°S to 90°N) for selected time periods of the simulated 2008/9 Eliassen-Palm (E-P) flux arrows ($m^2 s^{-2}$) to indicate wave propagation and contours of E-P flux divergence. The figure is identical to figure 3 but contours now show EP flux divergence ($ms^{-1} day^{-1}$) instead of zonal winds. Values of $-1ms^{-1}day^{-1}$ are shaded.

(a) 2008/9 AllTrop-UpStrat-Eq in which **u,v** and T were relaxed towards ERA-Interim data throughout the troposphere and additionally **u** was relaxed towards ERA-Interim data above 5 hPa and between 0-10°N. This simulation successfully reproduced the observed sudden warming event.
(b) 2008/9 AllTrop experiment, identical to AllTrop-UpStrat-Eq but without the additional relaxation above 5 hPa between 0-10°N. This simulation failed to correctly reproduce the timing of the warming event. (**c)** the difference between the two simulations (AllTrop-UpStrat-Eq minus AllTrop).

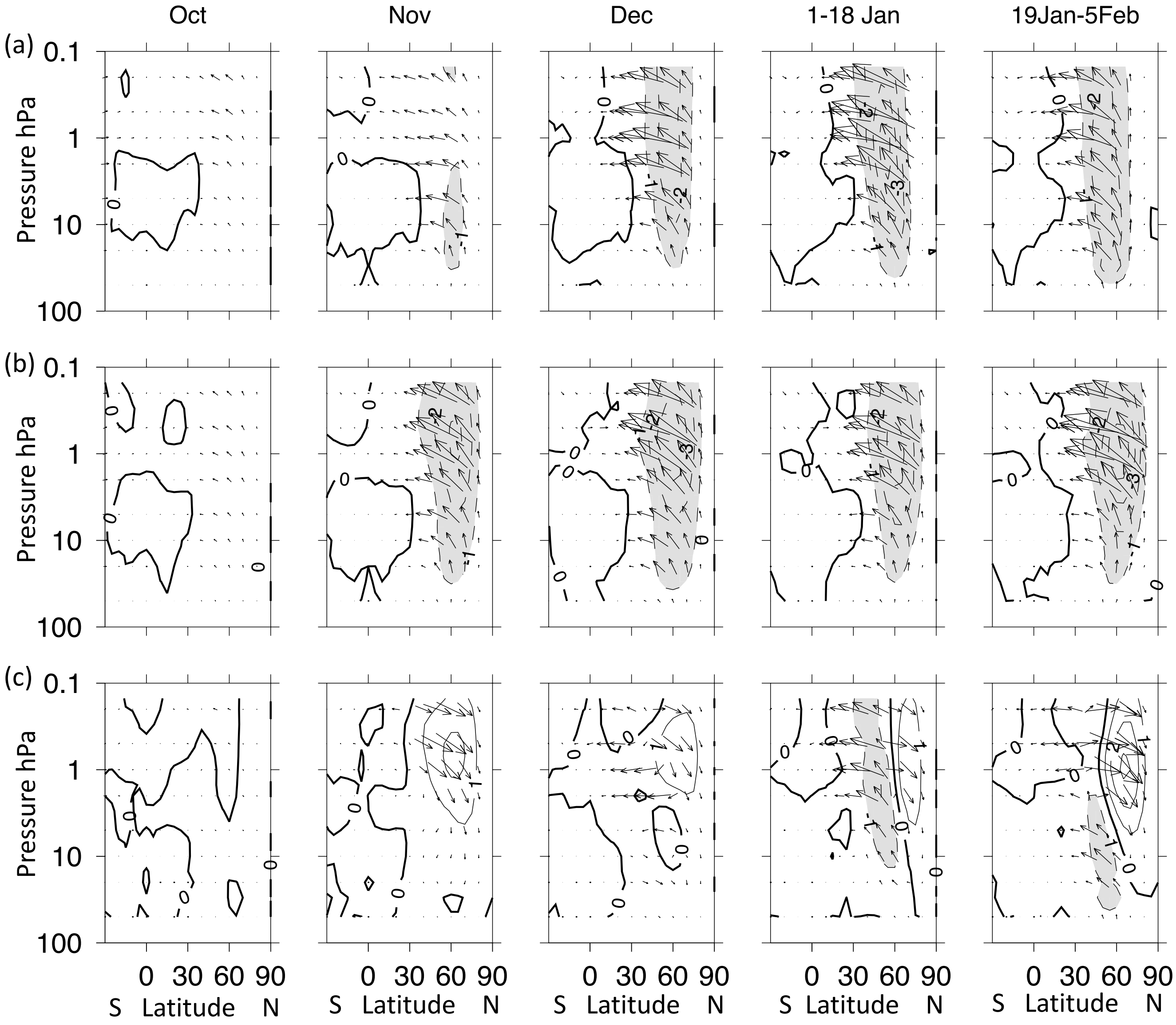

Supplementary Figure 5. 2008/9 Equatorial Winds. Time evolution of 2008/9 zonally averaged equatorial zonal winds (ms^{-1}) from selected simulations. **(a)** UpStrat-Eq-ERA, in which equatorial relaxation towards ERA-Interim zonal wind data was imposed above 5 hPa. **(b)** UpStrat-Eq-MERRA, as in (a) but with relaxation towards MERRA2 data **(c)** UpStrat-Eq-ERAClim, as in (a) but with relaxation towards ERA-Interim 1979-2018 climatology. (d) UpStrat-Eq-Clim40, as in (c) except that the ERA-Interim climatology has been modified by adding 40 ms^{-1} to more closely resemble satellite observations (see Supplementary Table 1 for details of the individual experiments).

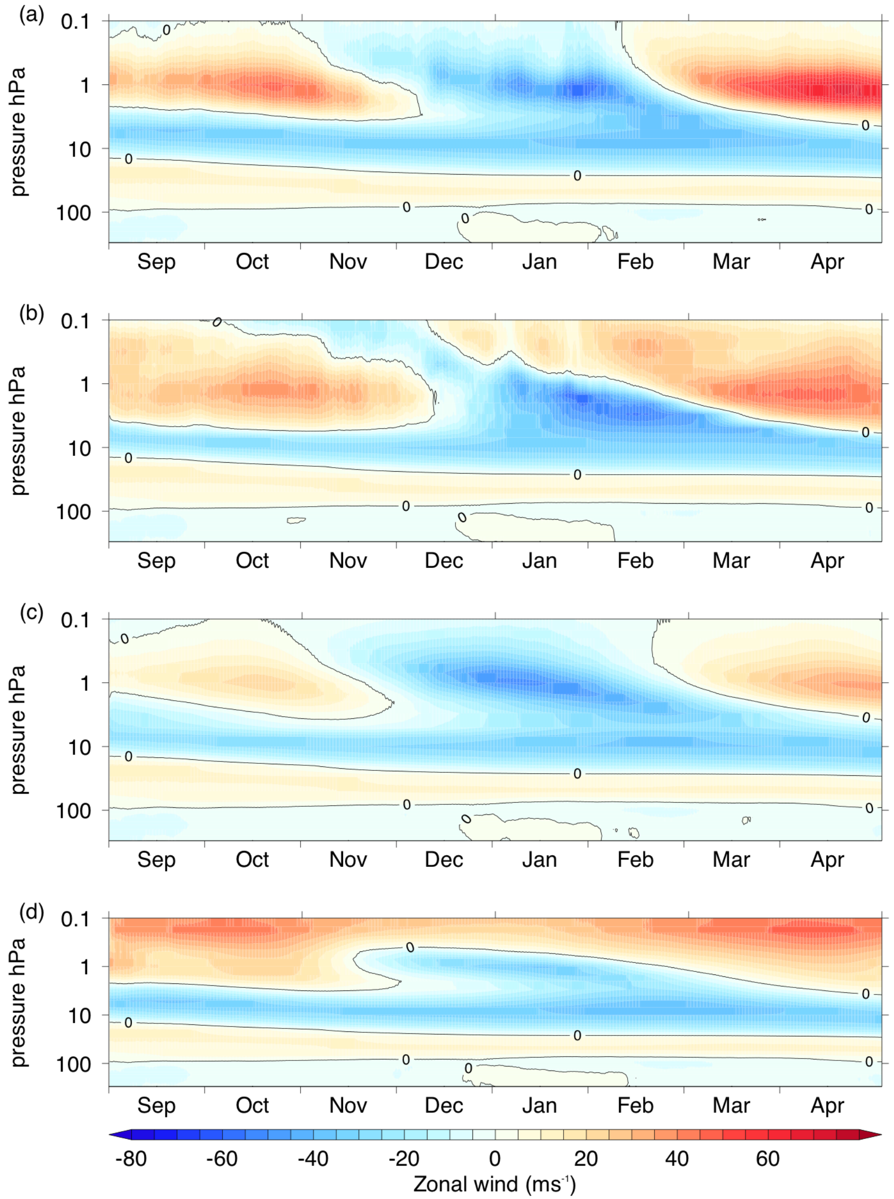

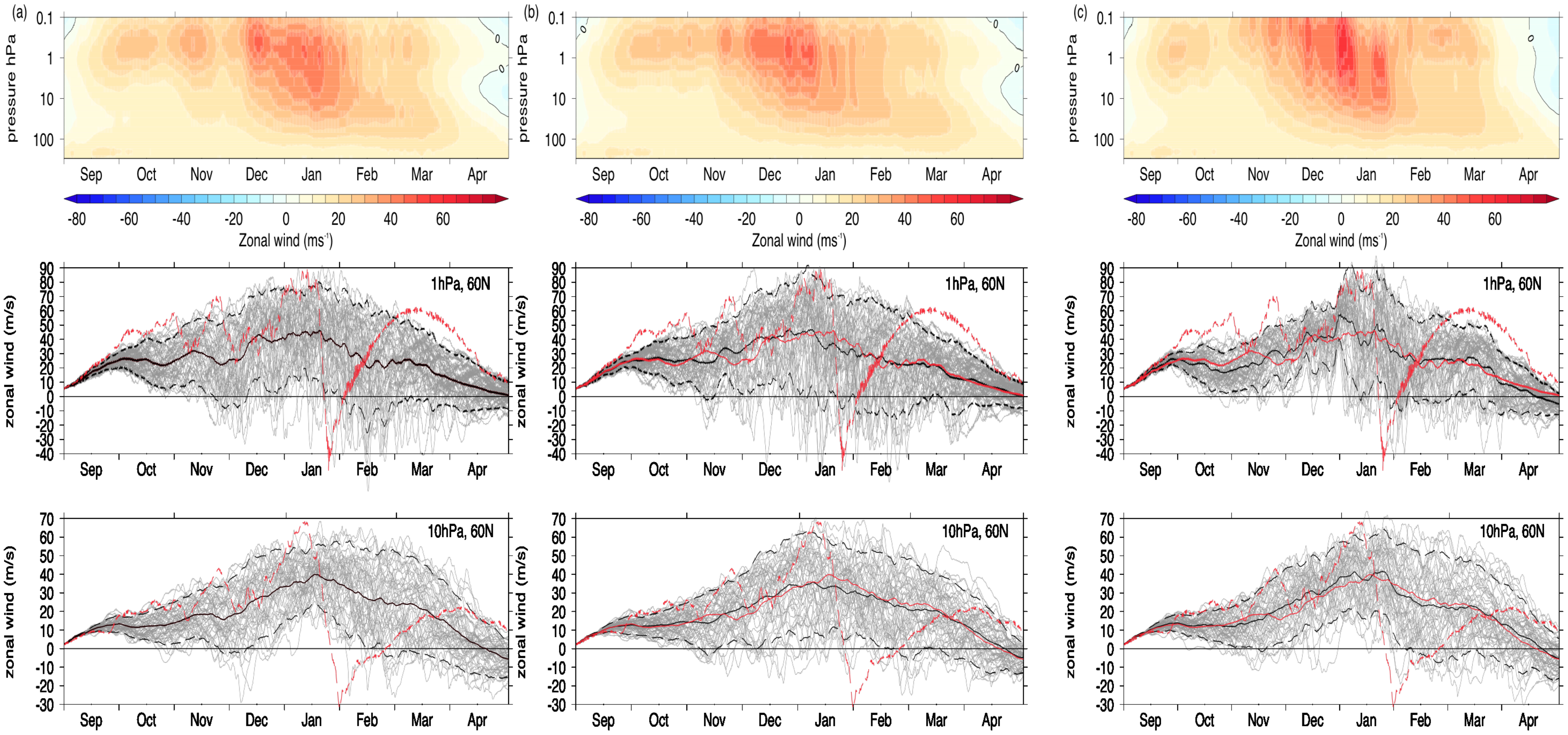

Supplementary Figure 6. 2008/9 UpStrat-Eq-ERA Experiments. 2008/9 evolution of zonally averaged zonal winds (ms^{-1}) at 60°N. **(a)** Control run, in which no relaxation was imposed. **(b)** UpStrat-Eq-ERA, in which relaxation towards ERA-Interim zonal winds **u** was imposed above 5 hPa and between 0-10°N. (**c)** UpStrat-Eq-ERA in which relaxation to ERA-Interim **u, v** and T was imposed above 5 hPa and between 0-30°N. Top rows show the ensemble-mean evolution while the lower panels show the evolution of individual ensemble members (grey lines) at selected pressure levels. Note that the selected pressure levels are different from those in Figure 2 and Supplementary Figures 1-3. Thick solid (dashed) black lines show the ensemble mean (1.5 standard deviation range). Dashed red lines show the ERA-Interim data. Red solid line shows the Control Run ensemble-mean for comparison.

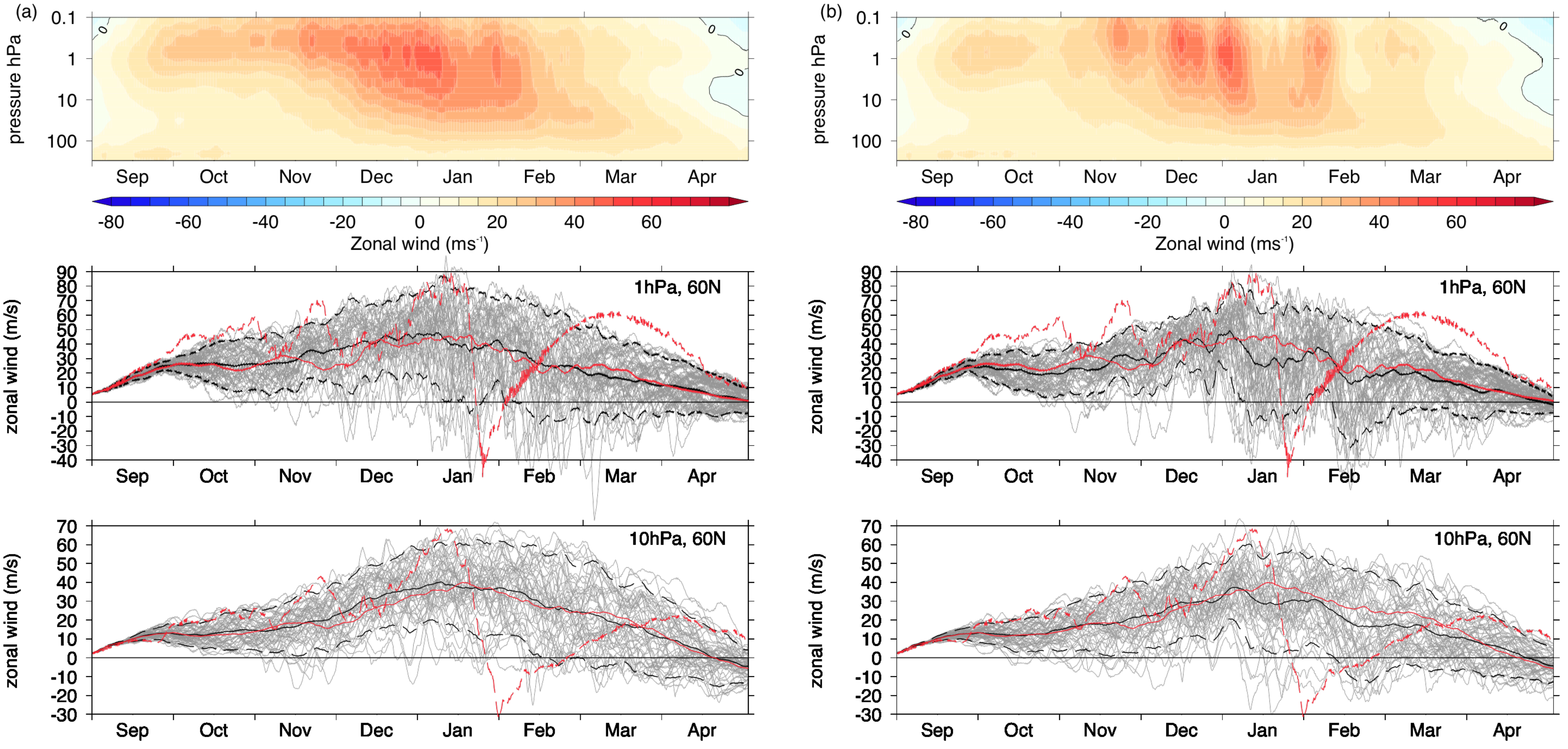

Supplementary Figure 7. 2008/9 UpStrat-Eq-MERRA Experiments. 2008/9 evolution of zonally averaged zonal winds (ms^{-1}) at 60°N. **(a)** UpStrat-Eq-MERRA, in which relaxation to MERRA2 zonal winds **u** was imposed above 5 hPa and between 0-10°N. (**c)** UpStrat-Eq-MERRA in which relaxation to MERRA2 **u, v** and T was imposed above 5 hPa and between 0-30°N. Top rows show the ensemble-mean evolution while the lower panels show the evolution of individual ensemble members (grey lines) at selected pressure levels. Note that the selected pressure levels are different from those in Figure 2 and Supplementary Figures 1-3. Thick solid (dashed) black lines show the ensemble mean (1.5 standard deviation range). Dashed red lines show the ERA-Interim data. Red solid line shows the Control Run ensemble-mean for comparison.

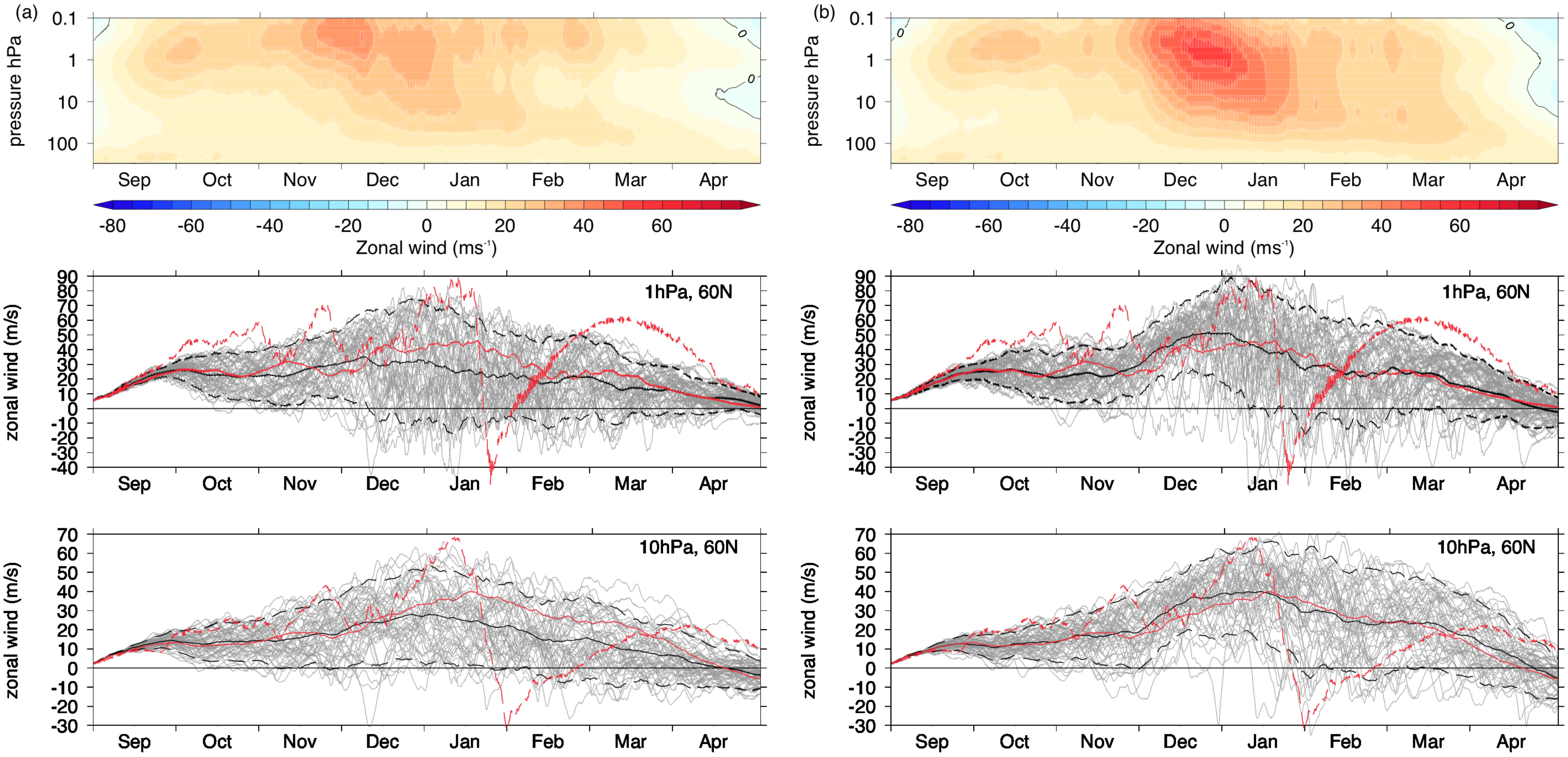

Supplementary Figure 8. Experiments relaxing towards ERA-Interim Climatology. 2008/9 evolution of zonally averaged zonal winds (ms^{-1}) at 60^oN. **(a)** the UpStrat-Eq-Clim experiment in which the **u, v** and T fields were relaxed to ERA Interim climatological values in the region $0\text{-}30^oN$ above 5 hPa. **(b)** UpStrat-Eq-Clim40 in which the zonal winds between $0\text{-}10^oN$ above 0.5 hPa were relaxed toward ERA Interim climatological values with 40 ms^{-1} added so that the SAO evolution more closely resemble satellite observations. Top rows show the ensemble-mean evolution while the lower panels show the evolution of the individual ensemble members (grey lines) at selected pressure levels. Note that the selected pressure levels are different from those in Figure 2 and Supplementary Figures 1-3. Thick solid (dashed) black lines show the ensemble mean (1.5 standard deviation range). Dashed red lines show the ERA-Interim data. Red solid line shows the Control Run ensemble-mean for comparison.

Supplementary Table 1. Summary of Experimental Results. Overview of results from the main experiments. First two columns provide the experiment name and a brief overview of experimental set-up. 3rd column shows the Pearson correlation coefficient between the December-January-February (DJF) averaged zonally-averaged zonal wind at 60°N, 10 hPa from the experiment compared with the corresponding evolution in the ERA-Interim data. The 4th and 5th columns show the number of identified stratospheric sudden warmings (SSWs) in DJF and within ±15 days of the observed SSW. Astericks in the 3rd, 4th and 5th columns indicate where the results show improvements over the corresponding Control Experiment and the differences between them are statistically significant at a level of 95% or greater (see Methods).

For each event the date of the observed SSW is provided together with an indication of the type of warming event, the phase of the quasi biennial oscillation (QBO) based on the sign of the ERA-interim equatorial zonal winds at 50 hPa (QBO-W or QBO-E) and the phase of the solar cycle based on the sunspot number (solar max or solar min).

Note that the number of SSWs identified in the 1988/9 AllTrop experiment is only 1 despite the 0.92 correlation. This is because although the evolution of the winds follows the observed fields reasonably well the SSWs did not quite penetrate to the 10 hPa level.

**Expt**	**Relaxation details**	**DJF corr**	**SSWs DJF**	**SSWs ±15 days**
**2008/9 (24th January 2009; split vortex; QBO-W; solar min)**				
**Control**	none	0.29	22	2
**AllTrop**	u,v,T from surface to tropopause at all lats	0.56*	49*	10*
**AllTrop-UpStrat-Eq**	u,v,T from surface to tropopause at all lats plus 2008/9 u above 5 hPa between 0-10N	0.98*	50*	50*
**UpStrat-Eq-Clim**	Climatological u,v,T above 5 hPa between 0-30N	0.77*	36*	19*
**UpStrat-Eq-Clim40**	Climatological u above 0.5 hPa between 0-10N with 40m/s added so that SAO evolution more closely resembled MLS and SABER data	0.75*	26	14*
**1988/9 (21st February 1989; split vortex; QBO-W; solar max)**				
**Control**	none	0.85	16	10
**AllTrop**	u,v,T from surface to tropopause at all lats	0.92*	1	1
**AllTrop-UpStrat-Eq**	u,v,T from surface to tropopause at all lats plus 1988/9 u above 5 hPa between 0-10N	0.98*	50*	50*
**UpStrat-Eq-Clim**	climatological u,v,T above 5 hPa between 0-30N	0.87	24	24*
**UpStrat-Eq-Clim40**	climatological u above 0.5 hPa between 0-10N with 40m/s added so that SAO evolution more closely resembled MLS and SABER data	0.82	14	12
**2005/6 (21st January 2006; displaced vortex; QBO-E; solar min)**				
**Control**	None	0.71	30	19
**AllTrop**	u,v,T from surface to tropopause at all lats	0.14	50*	50*
**AllTrop-UpStrat-Eq**	u,v,T from surface to tropopause at all lats plus 2005/6 u above 5 hPa between 0-10N	0.91*	50*	50*
**UpStrat-Eq-Clim**	climatological u,v,T above 5 hPa between 0-30N	0.90*	39	33*
**UpStrat-Eq-Clim40**	climatological u above 0.5 hPa between 0-10N with 40m/s added so that SAO evolution more closely resembled MLS and SABER data	0.11	30	11

Supplementary References

1. Baldwin, M.P., Birner, T., Brasseur, G., Burrows, J., Butchart, N., Garcia, R., Geller, M., Gray, L.J., Hamilton, K., Harnik, N., Hegglin, M., Langematz, U., Robock, A., Sato, K. & Scaife, A. 100 Years of Progress in Understanding the Stratosphere and Mesosphere. Chapter 20 in A Century of Progress in Atmospheric and Related Sciences: Celebrating the American Meteorological Society Centennial, AMS Meteorological Monographs (2019).
2. Matthewman, N.J. & Esler, J.G. Stratospheric sudden warmings as self-tuned resonances. Part I: Vortex splitting events. *J. Atmos. Sci.* **68**, 2481–2505 (2011).
3. O'Neill, A. & Pope, V.D. Simulations of linear and non-linear disturbances in the polar stratosphere. *Q. J. Roy. Met. Soc.* **114**, 1063–1110 (1988).
4. McIntyre, M.E. How well do we understand the dynamics of stratospheric warmings. *J. Meteorol. Soc. Jpn*. **60** (1), 37-65 (1982).
5. Charlton, A.J. & Polvani, L.M. A new look at stratospheric sudden warmings, Part I: climatology and modelling benchmarks. *J. Clim.* **20** (3), 449-469 (2007).
6. Butler, A.H., Seidel, D.J., Hardiman,, S.C., Butchart, N. & Birner, T. A Match Defining sudden stratospheric warmings. *Bull. Am. Met. Soc*. **1-16** (2015).
7. Butler, A.H., Sjoberg, J.P., Seidel, D.J. & Rosenlof, K. A sudden stratospheric warming compendium. *Earth System Science Data*, **9** (1), 63-76. Doi:10.5194/essd-9-63-2017 (2017).
8. Harada, Y., Goto, A., Hasegawa, H., Fujikawa, N., Naoe, H. & Hirooka T. A major stratospheric sudden warming event in January 2009. *J. Atmos. Sci*. **67** (6), 2052-2069 (2010).
9. Lida, C., Hirooka, T. & Eguchi, N. Circulation changes in the stratosphere and mesosphere during the stratospheric sudden warming event in January 2009. *J. Geophys. Res.* **119**, (12), 7104-7115. Doi:10.1002/2013JD021252 (2014).
10. Noguchi S, Mukougawa, H., Kuroda, Y., Mizuta, R., Yabu, S. & Yoshimura, H. Predictability of the stratospheric polar vortex breakdown: An ensemble re-forecast experiment for the splitting event in January 2009. *J. Geophys. Res.* **121**, 7, 3388-3404, doi:10.1002/2015JD024581 (2017).
