# Supplementary material for: Forecasting extreme stratospheric polar vortex events
Source: Nat Commun. 2020 Sep 15;11:4630. doi: 10.1038/s41467-020-18299-7 (PMC7492229; doi:10.1038/s41467-020-18299-7)
Supplement: Supplementary file 3 — Description of Additional Supplementary Files [file 41467_2020_18299_MOESM3_ESM.pdf]

Description of Additional Supplementary Files

File Name: Supplementary Movie 1
Description: The split vortex warming event of 2008/9. Polar stereographic animation of geopotential height fields (km) at 10 hPa for 2008/9 from the European Centre Reanalysis Interim (ERA-Interim) dataset showing the split vortex sudden stratospheric warming in January 2009. The movie starts from 15th December 2008 and has a time resolution of 24 hrs. The date progression can be seen above the movie.

File Name: Supplementary Movie 2
Description: Model simulation of the split vortex warming event of 2008/9. Polar stereographic animation of geopotential height fields (km) at 10 hPa for 2008/9 from the the AllTrop-UpStrat-Eq experiment in which the the winds and temperatures from the surface to the tropopause were relaxed toward the ERA-Interim data at all latitudes to capture the correct tropospheric wave forcing and additionally the zonal winds in the upper equatorial stratosphere between 0-10o N above 5 hPa were relaxed towards ERA-Interim data to capture the semi annual oscillation. The movie starts from 1st November 2008 and has a time resolution of 24 hrs. The date progression can be seen above the movie (note that the start date is 6 weeks earlier than in Supplementary Movie 1).
